# The Impact of TiO_2_ Nanoparticle Concentration Levels on Impulse Breakdown Performance of Mineral Oil-Based Nanofluids

**DOI:** 10.3390/nano9040627

**Published:** 2019-04-17

**Authors:** Ziyi Wang, You Zhou, Wu Lu, Neng Peng, Weijie Chen

**Affiliations:** 1 2011 Collaborative Innovation Center of Clean Energy and Smart Grid, Changsha University of Science & Technology, Changsha 410114, China; wzycsust@163.com (Z.W.); 15243694486@163.com (N.P.); chenweijie8558@163.com (W.C.); 2 College of Electrical Engineering, Shanghai University of Electric Power, Shanghai 200090, China; wuluee@shiep.edu.cn

**Keywords:** nanoparticle, oil insulation, breakdown, electron trapping, polarity effect

## Abstract

The insulation of mineral oil-based nanofluids was found to vary with different concentration level of nanoparticles. However, the mechanisms behind this research finding are not well studied. In this paper, mineral oil-based nanofluids were prepared by suspending TiO_2_ nanoparticles with weight percentages ranging from 0.0057% to 0.0681%. The breakdown voltage and chop time of nanofluids were observed under standard lightning impulse waveform. The experimental results show that the presence of TiO_2_ nanoparticles increases the breakdown voltage of mineral oil under positive polarity. The enhancement of breakdown strength tends to saturate when the concentration of nanoparticle exceeds 0.0227 wt%. Electronic traps formed at the interfacial region of nanoparticles, which could capture fast electrons in bulk oil and reduce the net density of space charge in front of prebreakdown streamers, are responsible for the breakdown strength enhancement. When the particle concentration level is higher, the overlap of Gouy–Chapman diffusion layers results in the saturation of trap density in nanofluids. Consequently, the breakdown strength of nanofluids is saturated. Under negative polarity, the electrons are likely to be scattered by the nanoparticles on the way towards the anode, resulting in enhanced electric fields near the streamer tip and the decrement of breakdown voltage.

## 1. Introduction

Nanofluid, i.e., base oil combined with nanoparticles, was firstly proposed by Choi from Argonne National Laboratory in 1995 [1]. V. Segal et al. first found that adding magnetic Fe_3_O_4_ nanoparticles into mineral oil not only increased the thermal conductivity, but also enhanced the insulating strength of oil [2]. The positive lightning impulse breakdown voltage of Fe_3_O_4_ nanofluid has nearly doubled compared with that of base oil, and the chop time increases from 12.0 μs to 26.0 μs. The ability of enhancing the thermal and dielectric performances of the base oil makes nanofluid a potential alternative to conventional mineral oil. Besides Fe_3_O_4_ nanoparticles, ZnO [3,4], ZrO [3], Al_2_O_3_ [3], SiO_2_ [4,5,6], GO [5], and AlN [7] nanoparticles were found to have the capacity to improve the insulating performance of oil. The performance of nanofluids not only depends on the type of nanoparticles, but also their concentration [8]. For example, in Lee et al.’s research [9], the AC breakdown of Fe_3_O_4_ nanofluids with volume concentration of 0.08% to 0.6% varies in the range of 38 kV to 42 kV. Madavan et al. found that, in the range of 0 to 0.25% volume fraction the AC breakdown voltage of Fe_3_O_4_, ZnO, and SiO_2_ nanofluids increased with the concentration of nanoparticles [4]. Ajay Katiyar et al. added commercially-purchased ZnO, ZrO_2_, and Al_2_O_3_ nanoparticles into the transformer oil. The AC breakdown voltage of nanofluids increased with the concentration of nanoparticles firstly and then began to decease with further higher concentration of nanoparticles [3].

TiO_2_ nanoparticles have already been extensively used in many fields such as environmental application, battery production, biomedical industry, personal care appliances, and cosmetics [10,11]. With the help of the surface modification method, TiO_2_ nanoparticles were found to be dispersed in mineral oil without agglomeration and precipitation for 18 months [12]. Breakdown strength of TiO_2_ nanofluids increases by 24% compared to that of base oil, and they have a good resistance to thermal aging and moisture deterioration [13,14,15].

The previous researches aimed at investigating effects of different nanoparticle types on the dielectric properties of insulating oil [2,3,4,5,6,7,8,9,10,11,12,13,14,15]. However, the mechanism behind the variation in breakdown voltages of nanofluids caused by different concentration levels was not investigated in detail. In addition, the effect of concentration level on dielectric properties of nanofluids were judged through AC breakdown tests in previous studies. However, AC breakdowns of oil are generally triggered by positive streamers bridging electrodes as positive streamers propagate much faster than negative ones in traditional mineral oil [16], and AC breakdown voltages are sensitive to impurity and moisture content in oil [17]. It is therefore difficult to evaluate the comprehensive insulating performance of nanofluids with only AC breakdown voltages, and to reveal the mechanism behind the breakdown performance of nanofluids with different concentration levels.

In this paper, the impacts of TiO_2_ nanoparticle concentration level on impulse breakdown performance of mineral oil were carefully investigated. Nanofluids combined with different weight percentages of TiO_2_ nanoparticles were prepared. Breakdown voltage and chop time of oil samples under standard lightning impulse voltages were measured. Eventually, the possible mechanism behind the modification of breakdown voltages caused by the change of TiO_2_ nanoparticle concentration level is discussed based on electron trapping theory.

The paper is structured as follows. Section 2 describes the preparation method of TiO_2_ nanofluids and the procedure of breakdown test. Section 3 exposes the breakdown characteristics of prepared samples under positive and negative lightning impulse voltages. A possible mechanism is proposed in Section 4 to explain the variation of breakdown performance of TiO_2_ nanofluids with different concentration Levels. The conclusions are presented in Section 5.

## 2. Experimental Descriptions

### 2.1. Sample Preparation

**Figure 1:**
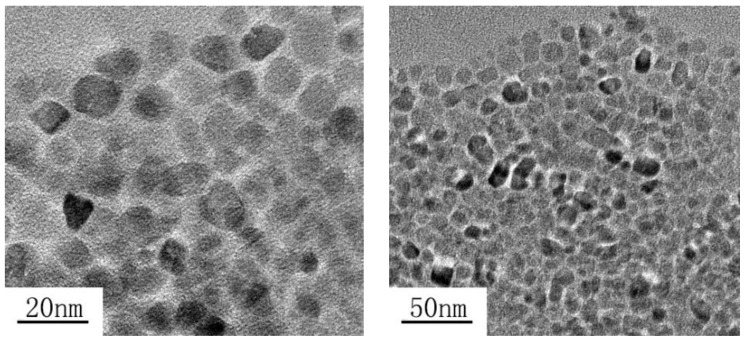
High-resolution transmission electron microscope (HRTEM) image of TiO_2_ nanoparticles.

TiO_2_ nanoparticles were prepared in laboratory by a solvothermal method. Titanium *n*-butoxide (CAS: 5593-70-4, Sigma-Aldrich, Shanghai, China) and DI water as reactants were firstly introduced into a mixed solution of cyclohexane (CAS: 110-82-7, Sigma-Aldrich, Shanghai, China) and triethylamine (CAS: 121-44-8, Sigma-Aldrich, Shanghai, China) under continuous stirring. After stirring for 5 min, oleic acid (CAS: 112-80-1, Sigma-Aldrich, Shanghai, China), acting as surfactant, was added into the above solution with vigorous agitation at room temperature (~25 °C). The successful mixture of acid and solution was subsequently heated at 150 °C for 24 h. The final product was then cooled down naturally and washed with distilled water and absolute ethanol (CAS: 64-17-5, Sinopharm Chemical Reagent Co., Ltd., Beijing, China) for few repetitions in order to remove the remained ions. The images of well-prepared TiO_2_ nanoparticles were observed by high-resolution transmission electron microscope (HRTEM, JEM-2100F, JEOL Ltd., Tokyo, Japan), as shown in Figure 1. It is shown that the TiO_2_ nanoparticles are in spherical shape and their average diameter is approximately 20 nm.

The mineral oil KI25X, produced in Karamay, China, was chosen as the base oil. A well-controlled prefiltered processing procedure was applied on the oil samples to remove the impurity particles. The filtered oil fulfilled the requirement of clean oil defined by CIGRE working group 12.17, i.e., the particle content with a diameter larger than 5 μm in the base oil is less than 300 per 100 mL. Samples of nanofluids with different concentration levels were prepared by suspending TiO_2_ nanoparticles, with weight percentages of 0.0057 wt%, 0.0114 wt%, 0.0227 wt%, 0.0454 wt%, 0.0681 wt%, and 0.0908 wt%, separately into clean base oil. The mixture process was carried out through ultrasonication for 10 min. An ultrasonic bath (KQ-700DB, Kunshan ultrasonic instrument CO. LTD., Kunshan, China) provided a stable ultrasonic energy (40 KHz, 500 W) to break apart the soft agglomerates between nanoparticles, and evenly disperse nanoparticles into base oil. Pure oil with no TiO_2_ nanoparticles contained was used as benchmark for comparison. Before breakdown tests, all oil samples were dried in vacuum oven with a pressure of below 100 Pa at 85 °C for 48 h to eliminate dissolved moisture and air bubble. The moisture contents in oil samples were ~5–8 ppm, which were measured by using a Metrohm 831 KF Coulometer (Metrohm AG. Herisau, Switzerland) according to Karl Fischer titration method.

### 2.2. Breakdown Test Procedure

**Figure 2:**
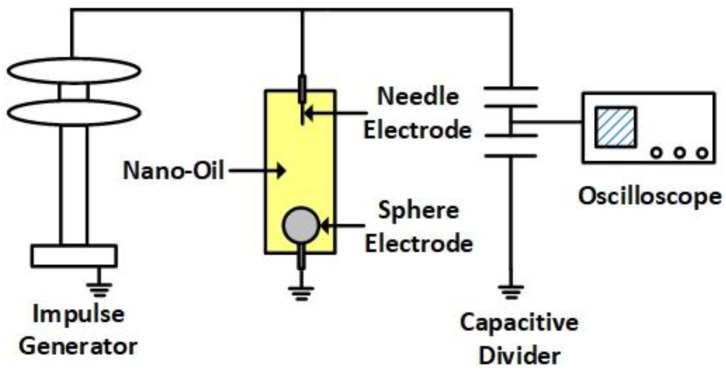
The sketch of test setup.

The lightning impulse breakdown tests of TiO_2_ nanofluids were carried out in a cylindrical oil container with a size of ϕ 40×195 mm. Approximately 250 mL oil sample was required for each breakdown test. The experimental setup was shown in Figure 2. A needle–sphere electrode system was used for the breakdown measurements. The tungsten needles with a tip radius of between 50 and 70 μm were chosen as the needle electrode. The spherical electrode is made of brass with a radius of 12.7 mm. A Marx impulse voltage generator was used to produce standard lightning impulsive voltage with ±1.2/50 μs waveform onto the test cell. Due to the widely observed polarity effect that the negative breakdown voltage is generally higher than the positive one at the same gap distance, the gap distance was set as 25 mm and 10 mm for breakdown tests under positive and negative polarities, respectively.

**Figure 3:**
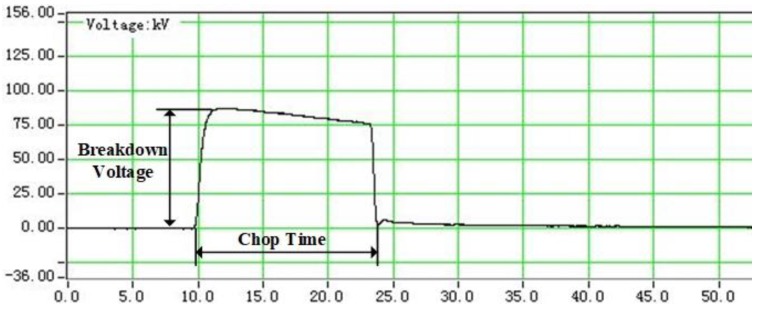
The breakdown voltage and chop time.

The step-by-step method following IEC 60897 was used for voltage rising. A selected proper value of impulse voltage was applied on the test cell initially. The voltage was applied three times consecutively. The time interval between each two successful pulses was 1 min. If breakdown did not happen, the voltage is increased by a step of 5 kV until breakdown occurs. A digital oscilloscope connecting with a capacitive voltage divider was used to measure the applied voltage waveform. When a breakdown occurs, the peak of applied voltage and the time when the wave abrupt dropped, as shown in Figure 3, are defined as the breakdown voltage and the chop time, respectively. At least 6 successful breakdowns for oil samples at each nanoparticle concentration level were obtained.

## 3. Experimental Results

### 3.1. Breakdown Characteristics under Positive Polarity

**Figure 4:**
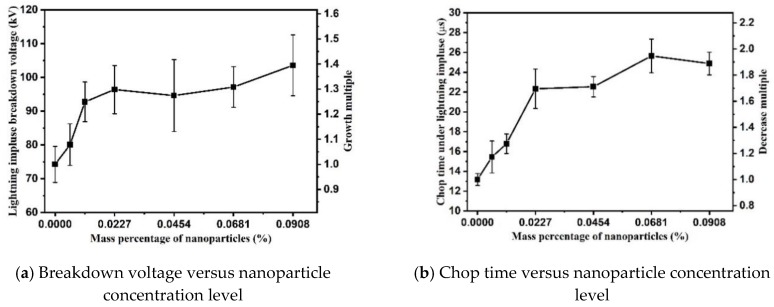
Positive impulse breakdown performance of TiO_2_ nanofluids.

The effects of TiO_2_ nanoparticle concentration level on positive breakdown performance are presented in Figure 4. It is shown that when the nanoparticle concentration level increases from 0 wt% to 0.0227 wt%, the positive breakdown voltage increases from 74.27 kV to 94.61 kV, i.e., the breakdown voltage increases by 27.38%. However, the breakdown voltage tends to saturate when the concentration level increases further. The positive breakdown voltage only increases 8.96 kV, i.e., from 94.61 kV to 103.57 kV, when the concentration level increases from 0.0227 wt% to 0.0908 wt%.

The changing trend of chop time is similar to that of breakdown voltage with only a slight difference. The longest chop time is 25.65 μs when the nanoparticle concentration level reaches 0.0681 wt%, which is 1.95 times higher than that of pure oil. It is noticed that no decreasing trend in both positive breakdown voltage and chop time is observed when the nanoparticle concentration level increases.

### 3.2. Breakdown Characteristics under Negative Polarity

**Figure 5:**
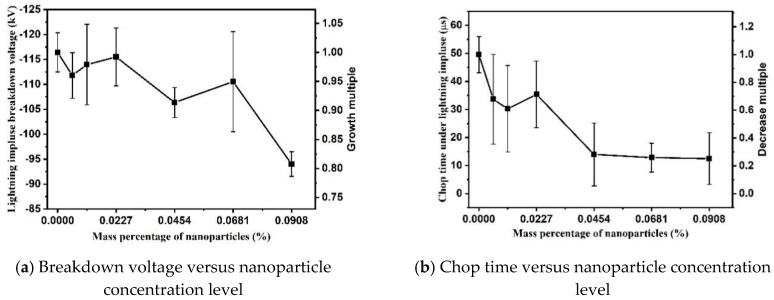
Negative impulse breakdown performance of TiO_2_ nanofluids.

As shown in Figure 5, the negative breakdown voltage of nanofluids decreases with the increase of concentration. The negative breakdown voltage of nanofluid at 0.0908 wt% is 94.00 kV, which is only 80.75% of that of pure oil, i.e., 116.41 kV. Meanwhile, the chop time of nanofluids under negative impulsive voltage reduces with the increase of nanoparticle concentration level. The chop time for pure oil is 49.78 μs on average under negative polarity, whereas the chop time is only 12.48 μs on average when particle concentration level is 0.0908 wt%. Compared with the breakdown characteristics under positive polarity, the data dispersion of breakdown characteristics is much larger under negative polarity. It might be caused by the splitting of electron propagating in different directions, and branching of streamers in highly nonuniform electric field when voltage polarity varies [18].

## 4. Discussion

The experimental results presented in Section 3 indicate that the breakdown characteristics of TiO_2_ nanofluids have important features:

(1) The presence of TiO_2_ nanoparticles increases the positive breakdown voltage and prolongs the chop time of the mineral oil-based nanofluids, whereas the breakdown voltage of oil slightly decreases with the presence of TiO_2_ nanoparticles. Define a ratio of average breakdown field strength r,

(1) $$r=\frac{E_{P}}{E_{N}}=\frac{V_{b.P}/D_{P}}{V_{b.N}/D_{N}}$$

where $V_{b.P}$ and $V_{b.N}$ represent the positive and negative breakdown voltage, respectively. $D_{P}$ and $D_{N}$ represent the gap distance used in the positive and negative breakdown tests, respectively. The ratio for TiO_2_ nanofluids, which is 0.334 at 0.0227 wt%, is higher than that for pure oil (0.255), indicating that the presence of TiO_2_ nanoparticles benefits the overall insulating properties of mineral oil.

(2) The positive breakdown voltage of TiO_2_ nanofluids increases gradually with the increase of nanoparticle concentration, until the concentration level reaches 0.0227 wt%. Beyond this threshold of particle concentration level, the increasing rate of breakdown voltage slows, and eventually the breakdown voltage levels off. An optimal value of TiO_2_ nanoparticle concentration level could be advised for industrial application based on the overall insulation performance of nanofluids.

The breakdown of mineral oil is the consequence of conductive pathways formed in bulk oil and bridge electrodes. These conductive pathways are named as ‘streamers’. Streamers have a filamentary or bushy structure depending on the test conditions, such liquid nature, voltage level, voltage polarity, and additives. In past decades, mechanisms behind the streamer propagation were extensively studied, but the conclusion is still pending [19]. It is accepted that streamers are formed by the ionization of oil molecules in high local field regions. Oil molecules are ionized into electrons and positive ions through field ionization, impact ionization, and photoionization processes [20,21,22]. Ionized electrons with higher mobility drift away from the ionization region quickly, leaving positive ions fall behind in the ionization region near streamer tips, and the propagating behavior of streamers is affected consequently. For positive streamers, the positive ions enhance the electric field strength in front of the streamer tips and induce further ionization in the oil gap. The propagation of positive streamers is facilitated and breakdown event is induced. For negative streamers, the positive ions cripple the electrical field strength of oil gap between the negative streamer tips and the anode. The propagation of negative streamer is restrained and a higher electrical stress is needed to trigger the breakdown. The differences in breakdown strength of insulating oil are generally called as the polarity effect of breakdown [23].

In order to explain the enhancement of positive breakdown voltage of transformer oil caused by presence of nanoparticles, J. George Hwang et al. [24] and Ricardo Albarracín et al. [25] proposed an electrodynamics model based on the charging dynamics of nanoparticles. In the electrodynamics model, polarized nanoparticles act as electron scavengers in nanofluid under electric stresses. They capture fast electrons and convert into negative charged nanoparticles. Charges on nanoparticles decrease the net charge density at streamer tips. The decrement of net charge homogenizes the electric field distribution at the streamer tips, leading to suppressed propagation of positive streamers. The electrodynamic processes involved in streamer propagation are closely related to the relaxation time constant of nanoparticles. The relaxation time constant *τ* is expressed as follows.

(2) $$\tau =\frac{2\varepsilon_{1}+\varepsilon_{2}}{2\sigma_{1}+\sigma_{2}}$$

where $\varepsilon_{1}$ and $\varepsilon_{2}$ represent the permittivity of base oil and nanoparticles, respectively. $\sigma_{1}$ and $\sigma_{2}$ represent the conductivity of base oil and nanoparticles, respectively. According to electron scavenger model, only the nanoparticles which can complete polarization within timescale of microseconds, could capture electrons and modify the propagation of streamers; the larger the time constant τ of nanoparticles, the less obvious the modification effect [25]. The electron scavenger model fits well with the enhancement of positive breakdown voltage in Fe_3_O_4_ nanofluids. However, it fails to explain the improvement in breakdown performance of nanofluids introduced by semiconductive TiO_2_ nanoparticles and nonconductive nanoparticles AlN [7] and SiO_2_ [4], whose relaxation time constants are much longer than the timescales involved in streamer propagation. Furthermore, according to the definition of electron scavenger model, the effect of nanoparticles on breakdown voltage of mineral oil should increase with nanoparticle concentration level. However, a saturation tendency on improvement of positive breakdown voltage is observed in this study.

**Figure 6:**
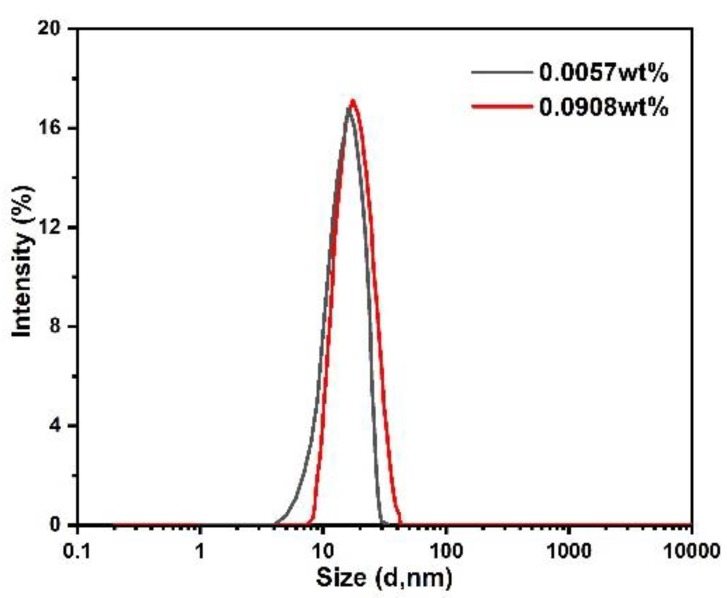
Size distribution of nanoparticles in TiO_2_ nanofluid.

Results in the literatures show that a decrement of breakdown voltage was generally found when the nanoparticle concentration level reaches a threshold value [26,27]. The researchers simply attributed the decrement of breakdown voltage to the agglomeration of nanoparticles at high concentration levels without any experimental verification. As the dispersion stability of nanoparticles in base oil directly affects the insulating performance of nanofluids [28], the size distributions of TiO_2_ nanoparticles in mineral oil is measured by Malvern ZS90 laser particle size analyzer based on the dynamic light scattering technology, as shown in Figure 6. The average diameter of TiO_2_ nanoparticles is stabilized at 20 nm when particle concentration level ranges are from 0.0056 wt% to 0.0908 wt%. When surface modified with oleic acid, TiO_2_ nanoparticles do not form particle clusters in base oil and the sizes of nanoparticles are still the same as that observed in Figure 1. This kind of TiO_2_ nanofluid has been reported to exhibit a high colloidal stability for more than 18 months at room temperature [12]. The high colloidal stability of nanoparticles indicates that the agglomeration of nanoparticles is not responsible for the saturation tendency of positive breakdown voltage with the increase of particle concentration level in this study.

**Figure 7:**
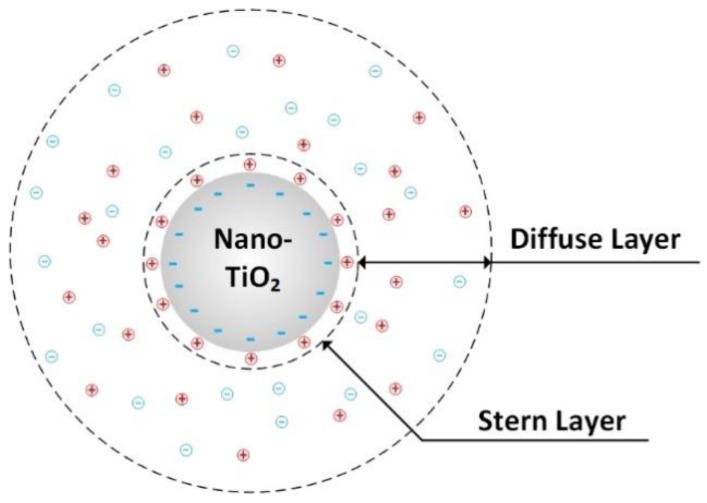
The sketch of electric double layer structure.

It is accepted that interface regions between nanoparticles and matrices become increasingly dominant in affecting the properties of composites as the particle size reduces to nanometric level [29]. When TiO_2_ nanoparticles are dispended into a base oil, they are charged due to the equalization of Fermi level or chemical potential; screening counter charges in surrounding oil phase will be established to confront the charges on particles. The screening counter charges are acquired through absorbing mobile ions and electrochemistry reorganization process in oil [30]. As a consequence, Electric Double Layers (EDLs) around the particles are formed, which are shown in Figure 7. The electrochemistry reorganization process includes both electronic polarizations and orientations of permanent dipoles in the interfacial region. These processes modify the collective polarization responses of surrounding molecules, which changes the electronic states and introduces more electronic traps in EDLs [31]. It is possible for electrons to be localized in these traps and drift with low mobilities ranging from 10^−7^ m^2^/Vs to 10^−8^ m^2^/Vs. These electrons cannot escape until they gain sufficient energy from thermal fluctuations and electric field [32]. The trapped electrons act as negative space charges in front of streamer tips. The net charge density is reduced, and the polarity effect in the streamer propagating process is distorted.

**Figure 8:**
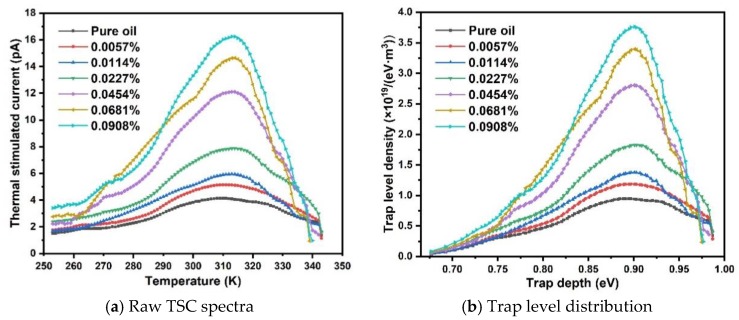
Thermal stimulated current (TSC) spectra and calculated trap level distribution of TiO_2_ nanofluid.

Trap characteristics in TiO_2_ nanofluids are obtained by analyzing thermal stimulated current (TSC) spectra according to a modified TSC theory [33], as shown in Figure 8.

**Figure 9:**
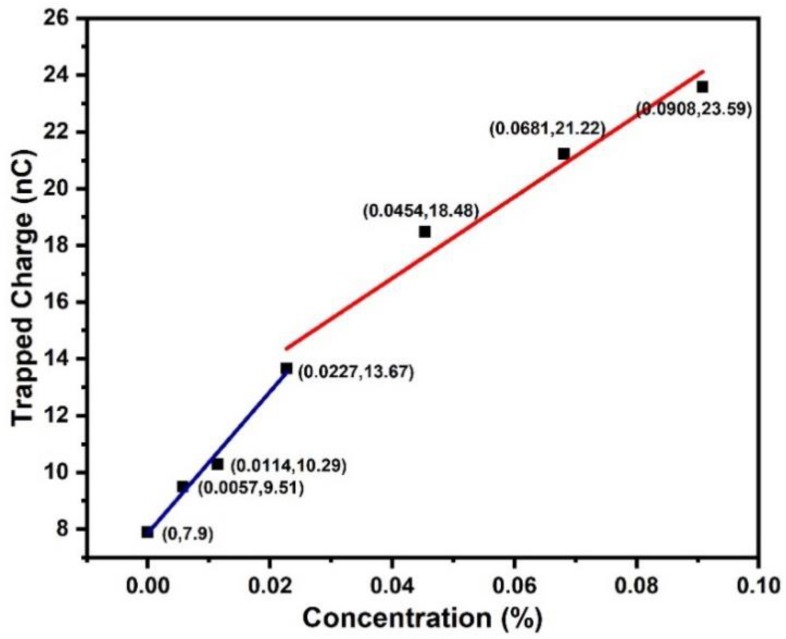
Amount of released charges versus particle concentration level.

As shown in Figure 8b, the trap level distribution exhibits a density peak at ~0.90–0.92 eV for all oil samples. Increasing the concentration level of TiO_2_ nanoparticles does not change the average depth of traps in nanofluid. However, the magnitude of trap density increases with the increase of nanoparticle concentration level, which seems to be inconsistent with the saturation tendency of breakdown voltage of nanofluids. In order to further study the trap characteristics in nanofluids, the total amounts of released charges are estimated by applying integration over the values of raw TSC spectra, as shown in Figure 9.

By fitting TSC curves piecewise, the charges quantity Q can be scaled as

(3) $$Q\left(n\right)=\{\begin{array}{c} 7.86+249.14n\\ 11.12+143.17n \end{array}\;\;\;\;\;\;\;$$

where n is the concentration level of nanoparticles in unit of wt%. The amount of released charges increases linearly with an increasing ratio of 249.14 when nanoparticle concentration level increases from 0 wt% to 0.0227 wt%. The increasing ratio drops to 143.17 when the nanoparticle concentration level is higher than 0.0227 wt%. It should be noticed that TSCs were obtained by measuring the current from fully polarized liquid samples with a constant rising temperature. Motions of charge carriers, i.e., de-trapping of localized charges and depolarization of dipolar molecules, are responsible for the measured currents. The incremental increase in concentration level of TiO_2_ nanoparticles, which have been polarized under DC electrical field, inevitably produces a higher depolarized current in TSC spectra. On the other hand, the reduced slope of released charges indicates the alteration of trap density in oil at higher nanoparticle concentration level, which is attributed to the overlap of interface region of nanoparticles.

Ideally, the nanoparticles are assumed to be homogeneously dispersed with diameter of 20 nm in spherical shape. The surface-to-surface distance *D* between the centers of neighboring nanoparticles can be estimated as follows [34].

(4) $$D={\langle \{\frac{\pi }{6}\left(\frac{\rho_{\mathrm{nano}}}{\rho_{\mathrm{oil}}}\right)\frac{100}{\mathrm{wt}\%}\left[1\;-\;\frac{100}{\mathrm{wt}\%}\left(1\;-\;\frac{\rho_{\mathrm{nano}}}{\rho_{\mathrm{oil}}}\right)\right]\}}^{\frac{1}{3}}-1\rangle d$$

where wt% presents the weight concentration of nanoparticles. $\rho_{nano}$ and $\rho_{oil}$ are the density of nanoparticles and transformer oil, respectively. d is the average diameter of nanoparticles. Interparticle distance calculated from Formula (4) is shown in Table 1.

The interparticle distance is ~239 nm when the diameter of nanoparticles is 20 nm and particle concentration level is 0.0227 wt%. As mentioned above, the trap states are induced by the polarization and local reorganization processes in EDLs. EDLs consist of two regions: the inner layer and the diffuse layer. The inner layer is defined by the Outer Helmholtz Plane, with a thickness of one or two molecular diameters. The thickness of diffuse layer, namely the Gouy–Chapman diffuse layer, is determined by the ionic strength, and scales more than hundreds of nanometers in insulating dielectrics [29]. When the concentration level of nanoparticle is higher than 0.0227 wt%, the Gouy–Chapman diffusion layers of nanoparticles begin to overlap. As a result, the interfacial volume, as well as the quantity of traps in oil, stop to increase when higher concentration level of nanoparticles is reached.

**Figure 10:**
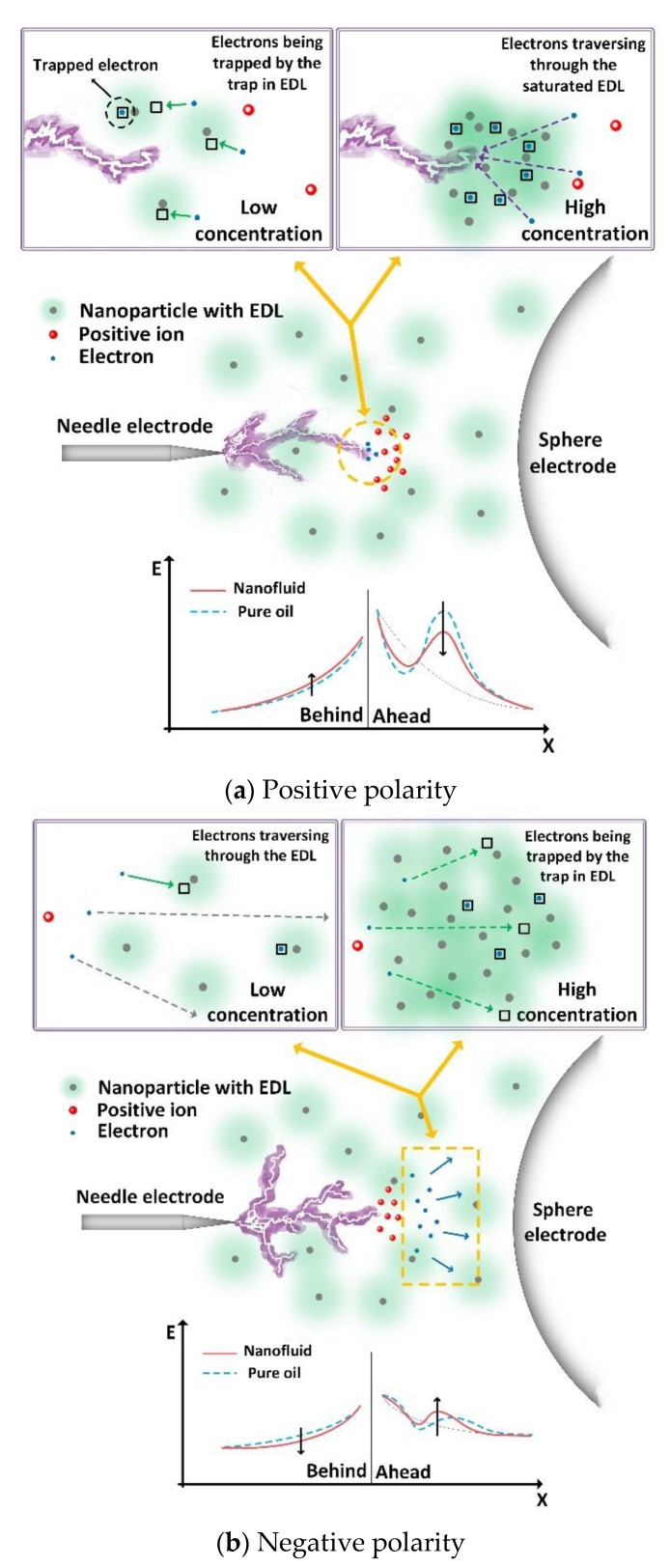
Streamer propagation model for indicating the effect of TiO_2_ concentration level on breakdown characteristics of nanofluid.

Based on above analyses, a model for describing the mechanisms behind the effect of TiO_2_ concentration level on breakdown characteristics of nanofluid oil is emerged, as shown in Figure 10. Semiconductive TiO_2_ nanoparticle, whose conductivity is in the range of 10^−2^ to 10^−10^ S/m depending on the physical and chemical structure [35,36], has a large relaxation time constant (10^−7^–10^1^ s). Thus, the evolution of breakdown voltage is not attributed to the polarization of TiO_2_ nanoparticles, because TiO_2_ nanoparticles are hard to establish their polarization at the time scale involved in streamer propagation to influence the electronic behavior in oil. The interfacial regions, which are attributed to the EDLs around nanoparticles, are responsible for the formation of electronic traps. The ionized electrons are able to be trapped when they pass through these interfacial regions. The trapped electrons act as negative space charges to reduce the net density of space charges at streamer tips. As a result, the polarity effect of breakdown is reduced.

The impact of electronic traps on breakdown strength of mineral oil is closely related to the interfacial region volume in oil. Interface regions in nanofluids increase with the increase of nanoparticle concentration level, resulting in more electronic traps to modify the breakdown strength of nanofluids. When interfacial regions of nanoparticles become to overlap with each other, the interfacial region volume and trap density in oil stop to increase any more. The electrons are ionized under high local electric stress in front of streamers. As ionized electrons centralized toward the positive streamer tip, the maximum quantity of trapped electrons is then limited by the local trap density near streamer tip. Once the interfacial region overlaps with each other, quantity of trapped electrons becomes level off, even as concentration level of nanoparticles increases further. Consequently, the saturation tendency of positive breakdown voltage is observed. Under negative polarity, the electrons are injected from the negative streamer tip and split into bulk oil. The trapped electrons are more sporadic in nanofluids and less restricted by the local trap density. Besides, the scatter probability of electrons increases with the suspense of nanoparticles. The scatted electrons are easier to be trapped on their travel path. As a result, the negative breakdown voltage of nanofluid decreases with the further increase of nanoparticle concentration level.

## 5. Conclusions

The impacts of nanoparticle concentration level on the breakdown characteristics of mineral oil-based nanofluids were investigated in this study. The breakdown voltage and chop time of nanofluid samples were measured under positive and negative lightning impulsive voltages. Experimental results show that the polarity effect on breakdown characteristics of nanofluids, caused by accumulated space charges around streamer tips, is reduced by suspending TiO_2_ nanoparticles. Under positive polarity, the insulating strength of nanofluid increases linearly with the concentration level of TiO_2_ nanoparticles, until the concentration level reaches the threshold value of 0.0227 wt%. Under negative polarity, insulating strength of nanofluid gradually decreases with the increase of particle concentration level. Prior work suggested that the insulation modification of nanofluids is caused by the electron scavenger effect of polarized nanoparticles. However, the electron scavenger model fails to explain the modification in breakdown performance of TiO_2_ nanofluids as the large relaxation time constant of semiconductive TiO_2_ nanoparticles. In this paper, the reduced polarity effect is attributed to the induced electronic traps at the EDLs of nanoparticles. The electrons are trapped in the EDLs of nanoparticles and reduce the net charge density in front of streamers. When the nanoparticle concentration level reaches 0.0227 wt% or higher the Gouy–Chapman diffusion layers begin overlap, resulting in the cessation of increase in trap density of oil. The number of trapped electrons in front of positive streamer is limited by trap density in nanofluids at higher concentration level. The gradual decrease of negative breakdown voltages is possibly induced by the increased trapped electrons in the oil gap, due to the increasing divergence of nanoparticle distributions. Although the model explains the breakdown voltages variation of TiO_2_ nanofluids, but it is proposed based on the TSC results in this study. Whether it is suitable to explain the insulation of other types of nanofluids requires further researches. Besides, the model did not consider the effect of surfactant on the trap characteristics in nanofluids. Future work should therefore include studying the relationship among different surfactants, trap and streamer propagation characteristics in nanofluids.

## Figures and Tables

**Table 1:** Distance between nanoparticles in transformer oil.

Concentration (wt%)	Interparticle Distance (nm)
0.0057%	386
0.0114%	304
0.0227%	239
0.0454%	188
0.0681%	160
0.0908%	147
